# Editorial: The role of nitrate in plant response to biotic and abiotic stress

**DOI:** 10.3389/fpls.2023.1240256

**Published:** 2023-07-06

**Authors:** Anis M. Limami, Bertrand Hirel

**Affiliations:** ^1^Univ Angers, Institut National de Recherche pour l'Agriculture et l'Environnement (INRAE), Institut de Recherche en Horticulture et Semence (IRHS), Angers, France; ^2^Institut Jean-Pierre Bourgin (IJPB), Institut National de Recherche pour l'Agriculture et l'Environnement (INRAE)-AGRo Paris Tech, Centre National de la Recherche Scientifique (CNRS), Versailles, France

**Keywords:** nitrate uptake, nitrate signaling, symbiosis, stress, NRT1(PTR), NRT2, NPF family

The interplay between mineral nitrogen (N) supply, carbon (C) assimilation and allocation of assimilates to sink organs is a finely regulated biological process as it allows plants to adapt to variations in nutrients availability.

Lepetit and Brouquisse tackled such biological process in a review on the control of nodule organogenesis, mature nodule functioning, and nodule senescence as a function of N demand *via* systemic signaling. These authors proposed a hypothetical model in which C resource allocation to the nodule is perceived as a systemic signal that tunes both the establishment and functioning of a plant/*Rhizobium* symbiosis. This model considers the plant N status, as rapid variations of the nodule sugar content are observed when there is a shift from N-satiety to N-deficit. The plant symbiotic capacities are adjusted to mineral N resources availability 1) through systemic N-satiety signaling that lowers the nodule sucrose content what triggers its senescence 2) through systemic N-deficit signaling (low level of mineral N or impairment of symbiosis) that increases sucrose content in the nodule thus stimulating its expansion and at the same time N foraging by the symbiotic root system.

Guo et al. studied C and N relationship in the context of rice grain as a C sink. They challenged the relation between N supply and C allocation to the grain in relation to chalkiness. This disorder is observed in deficient amyloplasts of the endosperm due to loosely packed starch granules. Increasing N application from 75 (N1) to 150 (N2) and 225 (N3) kg.ha-1 resulted in an increase in the starch content along with an accelerated grain development. Carbon partitioning between inferior and superior grains in the ear was improved resulting in optimized uniformity and overall reduction in chalkiness.

Besides its nutritional role nitrate is sensed by plants as a signal molecule involved in at least two types of actions: 1) a short-term signaling role named primary nitrate response (PNR) that results in the transcriptional regulation of hundreds of genes only a few minutes after its application, and 2) a long-term signaling role highlighted by its effect on root architecture *via* local and systemic signaling. Several nitrate transporters were discovered and described in various species. Depending on their affinity to nitrate, transporters belong to one of the two families: NRT1 renamed NPF (low affinity) and NRT2 (high affinity). Interestingly, it was found that nitrate transporters are also involved in nitrate signal mediation. In this Research Topic several new nitrate transporters are described and their roles as transporters and sensors are characterized. Wu et al. functionally characterized *NtNRT1.1B* in a heterologous system namely the nitrate-uptake defective yeast *HpDynt1*, in which the plant NtNRT1.1B enabled growth recovery. Cellular and tissue localization showed that NtNRT1.1B is targeted to the plasma membrane and that the cognate gene is expressed in the root stele and shoot–stem vascular tissues. Such findings suggest that NtNRT1.1B plays a role in long distance transport. Transgenic tobacco overexpressing NtNRT1.1B exhibited higher biomass, which allowed the authors to propose that this gene can be a target for improving N-use efficiency.

By using bioinformatics and RNA-seq Cheng et al. identified 92 and 88 putative NPF genes from *Setaria italica* L. and its wild ancestor *Setaria viridis* L. respectively. *SiNPF* gene family exhibited high number of duplication events. The authors identified 26 tandem duplications and 13 segmental duplications. The response of *SiNPF* gene family to low nitrate stress was analyzed by a series transcriptomic analysis performed during plant growth. This approach suggested that the tandem *SiNRT1.1B.1* and *SiNRT1.1B.2* from *Setaria italica* L. (Foxtail millet) could contribute to low N tolerance in this species. Functional characterization of both genes in Yeast and Arabidopsis by complementation of mutants confirmed their nitrate transport activity.

In the legume *Lotus japonicus* Rogato et al. characterized and described the role of a high affinity nitrate transporter, *LjNRT2.1*, in the response to changes of N availability in the surrounding environment. Characterization of two *Ljnrt2.3* knock out mutants showed that LjNRT2.3 fulfils a nutritional and signaling role as it is involved in root nitrate acquisition and signaling controlling lateral root elongation in response to N starvation. This characterization also suggested an epistatic interaction between *LjNRT2.3* and *LjNRT2.1* as the induction of *LjNRT2.1* when nitrate availability is low was abolished in *Ljnrt2.3* mutant.

Vacuoles are important storage compartments for nitrate. They allow the cell to cope with an excess nitrate when its uptake exceeds N utilization and mobilization when plants N demand is not met by N availability. Lu et al. work is dedicated to the tonoplast-localized nitrate transporters, namely NPF5.10, NPF5.14 and NPF8.5. Expression of the corresponding genes in *Xenopus laevis* oocytes confirmed their nitrate transport activity. Overexpression of these genes in Arabidopsis reduced nitrate content in the vacuoles supporting their role in vacuolar nitrate efflux. Interestingly the six genes belonging to the NPF family (NPF5.10, NPF5.14, NPF8.5, NPF5.11, NPF5.12, and NPF5.16) were all induced by mannitol. Furthermore, nitrate accumulated more in the sextuple mutant than that in the wild type after mannitol treatment. Altogether these findings allowed authors to propose that nitrate efflux from the vacuole to the cytosol could contribute to plants response to osmotic stress rather than to mineral N nutrition.

In order to get better insight on the complex mechanism of genes regulation by nitrate in apple (*Malus domestica*) Wen et al. identified the function of the transcription factor (TF) MdHHO3 ((HYPERSENSITIVITY TO LOW PHOSPHATE-ELICITED PRIMARY ROOT SHORTENING1 HOMOLOG 3) a member of the GARP family. These authors showed that MdHHO3 inhibits genes transcription by binding to a motif-containing GAATC present in their promotor. Accordingly, authors showed that MdHHO3 negatively regulated the expression of the nitrate transporter MdNRT2.1 by binding to its promotor. Characterization of Arabidopsis and tobacco overexpressing MdHHO3 showed that the transcription factor controls a large portfolio of genes related to nitrate nutrition. In MdHHO3 overexpressors, nitrate transport-related genes were down-regulated and nitrate assimilation-related genes were upregulated. Furthermore, senescence was induced in Arabidopsis and tobacco overexpressing MdHHO3 under nitrate deficiency.

## Author contributions

AL wrote the first draft that was reviewed and finalized by AL and BH. All authors contributed to the article and approved the submitted version.

